# Sulfometuron-Methyl Disrupts Early Zebrafish Embryonic Development via Wnt/β-Catenin Signaling Pathway

**DOI:** 10.3390/toxics14090835

**Published:** 2026-09-20

**Authors:** Xiaomei Zhang, Jinlong Liu, Yongxin Guo, Jun Zhong, Yan Dong, Shuoqian Cheng, Ziwei Li, Weidong Qiang, Huiyan Wang

**Affiliations:** 1 School of Pharmacy, Jilin Medical University, Jilin 132013, China; 2 National Engineering Research Center of Cell Growth Factor Drugs and Protein Preparations (Jilin Center), Jilin 132013, China; 3 College of Medicine, Huanghuai University, Zhumadian 463000, China; 4 Zhumadian Key Laboratory of Zebrafish-Intelligent Pharmaceutical Technology Collaborative Innovation, Zhumadian 463000, China

**Keywords:** sulfometuron-methyl, zebrafish, embryonic developmental toxicity, Wnt/β-catenin signaling pathway

## Abstract

Sulfometuron-methyl (SM), as a sulfonylurea herbicide, is widely used in agricultural production. In recent years, it has also been employed in the control and management of Spartina alterniflora in some coastal areas of China. Its potential risks to aquatic ecosystems have drawn increasing attention. However, the current data on the systematic toxicity assessment of SM in model organisms is still very limited. This study used zebrafish (*Danio rerio*) as an in vivo model to systematically evaluate the toxic effects of SM on early embryonic development and focused on the mediating mechanism of the Wnt/β-catenin signaling pathway in it. Zebrafish embryos were exposed to different concentrations (10, 20, 40 mg/L) of SM starting from 1.75 hpf until 72 hpf. The results showed that SM significantly increased the embryo mortality rate, decreased the hatching rate, and induced developmental malformations such as shortened body length and yolk sac edema in a concentration-dependent manner. At the mechanism level, SM exposure significantly reduced the number of H3P-positive mitotic active cells in the embryos, suggesting that fewer cells entered mitosis; meanwhile, the activities of superoxide dismutase (SOD) and catalase (CAT) showed compensatory changes, indicating that oxidative stress was induced in the embryos, which may contribute to subsequent cellular damage. qRT-PCR analysis further revealed that SM exposure downregulated the transcriptional levels of cell cycle-related genes (*CyclinD1*, *CDK4*, *CDK6*), while upregulating the expression of apoptosis-related genes (*p53*, *bax*, *caspase-9*, *caspase-3*), suggesting that cell cycle arrest and the dysregulation of the endogenous apoptotic pathway were activated. Additionally, SM exposure could dysregulate the Wnt/β-catenin signaling pathway, specifically manifested as significant upregulation of target genes *C-myc* and *Ctnnb2*, and significant downregulation of the negative regulatory factor *Axin2*. Using the Wnt/β-catenin pathway-specific inhibitor IWR-1 for intervention could partially reverse the embryonic developmental retardation phenotypes induced by SM, providing additional evidence for the mediating role of this pathway in the developmental toxicity of SM. In summary, this study systematically elucidates the multi-level molecular mechanism by which SM induces oxidative stress and cell apoptosis through the abnormal dysregulation of the Wnt/β-catenin signaling pathway, reduced mitotic activity, and ultimately drives the toxicity of zebrafish embryo development. This provides new experimental evidence and a theoretical basis for the aquatic ecological risk assessment of SM.

## 1. Introduction

Sulfometuron-methyl (SM) is a sulfonylurea herbicide that is widely used in agricultural production for the control of broadleaf and grassy weeds [1]. Its mechanism of action lies in selectively inhibiting the activity of acetolactate synthase (ALS), thereby blocking the biosynthesis of branched-chain amino acids such as valine, leucine, and isoleucine, and ultimately achieving the herbicidal effect [2]. Since the advent of sulfonylurea herbicides in the 1970s, these compounds, due to their broad-spectrum herbicidal activity, excellent selectivity, and low application dosage, have become an indispensable part of the global agricultural chemical market. However, SM has a long residual period in soil, with a half-life of approximately 28 days (depending on environmental conditions), and its high water solubility gives it the potential to migrate to aquatic environments [3]. In recent years, SM has been used in some coastal areas of China for the control of the invasion of Spartina alterniflora [4]. After application, SM can enter adjacent aquatic ecosystems through surface runoff, rain leaching, and sedimentation, thereby posing a potential exposure risk to non-target aquatic organisms. Environmental monitoring studies have reported detectable concentrations of SM in surface waters, with levels reaching up to 0.020 μg/L in certain regions [5]. While such concentrations are substantially lower than the experimental doses used in toxicological studies, they underscore the potential for SM to enter aquatic ecosystems and warrant systematic investigation of its toxicity profiles [6]. Studies have shown that SM exposure can significantly alter the composition and function of soil microbial communities, thereby affecting the health and stability of the local ecosystem [7]. Although the existing literature has reported the residue behavior and degradation dynamics of SM in soil and crops, systematic research on its toxic effects on aquatic organisms, especially the early embryonic stage of fish, and its molecular mechanisms is still extremely scarce. Therefore, assessing the toxic effects of SM on aquatic organisms has important environmental and health significance.

Zebrafish (*Danio rerio*), as a mature vertebrate model, has been widely used in toxicological research. This model has many unique experimental advantages, including optical transparency of embryos, in vitro development, short reproductive cycle, large single egg-laying capacity, and high homology of the genome with that of humans [8,9]. Among them, the transparency of the embryo enables researchers to track morphogenesis and cellular dynamics in real time during the early development process, providing an ideal operational platform for the in vivo assessment of chemical developmental toxicity [10]. In recent years, zebrafish models have been widely used in research fields such as developmental toxicity, immunotoxicity, neurotoxicity and cardiotoxicity of environmental pollutants [11]. Previous studies have shown that exposure to multiple herbicides can induce toxic effects such as death, delayed hatching, developmental malformations (such as edema, shortened body length, spinal curvature) and oxidative stress in zebrafish embryos [12]. For example, pyrazosulfuron-ethyl can cause delayed yolk absorption in embryos, delayed head development and ocular deformities [13]. Imazosulfuron can induce acute death within 48 h and cause deformities such as pericardial edema and spinal curvature [14]. The above findings suggest that sulfonylurea and representative herbicides have potential embryotrophic toxicity.

Wnt/β-catenin signaling pathway is one of the core pathways regulating vertebrate embryonic development, which plays an irreplaceable role in key processes such as dorso-ventral axis specialization, hematopoietic stem cell maintenance, organogenesis, and cell proliferation and differentiation [15]. In the stage of gastrulation in zebrafish, Wnt signaling is involved in basic developmental events such as anterior and posterior axis patterning and neuralization, and its activity is precisely regulated in time and space [16]. Abnormal dysregulation or inhibition of this pathway can lead to severe developmental defects. Studies have found that there is a genetic interaction between PGE2 and the Wnt signaling pathway to jointly regulate the developmental specification and regeneration of stem cells [17]. It has been reported that Wnt9a is required for the expansion of newborn hematopoietic stem cells in the aortic region [18]. In recent years, increasing evidence has shown that environmental pollutants can induce developmental toxicity by interfering with the Wnt signaling pathway. For example, environmental antibiotic exposure induces developmental toxicity in zebrafish embryos through Wnt/β-catenin signaling pathway dysregulation, manifested as pericardial edema, oxidative stress and impaired cardiac function. Tetrachlorantraniliprole induces neurodevelopmental toxicity through oxidative stress-mediated apoptosis and Wnt signaling pathway dysregulation [19]. Benzophenone exposure can induce cardiac developmental toxicity by up-regulating Wnt signaling [20]. Diclofop-methyl, a herbicide, can cause abnormal cardiac development by down-regulating the Wnt signaling pathway [21]. The above studies show the universality of Wnt signaling as a key target for developmental toxicity of environmental pollutants; however, direct evidence is still lacking on whether SM affects early embryonic development through this pathway.

In this study, we used zebrafish as an in vivo model to systematically evaluate the toxic effects of SM on early embryonic development, with a focus on the mechanism of the Wnt/β-catenin signaling pathway. The experiments included systematic assessment of embryonic developmental phenotype after SM exposure, detection of the number of mitotically active cells, determination of oxidative stress levels, analysis of apoptosis signals, and quantitative analysis of cell cycle and apoptosis-related gene transcription levels. At the same time, the specific inhibitor of the Wnt/β-catenin pathway, IWR-1 was used for functional rescue experiments to verify the key mediating role of WNT/β-catenin pathway in the developmental toxicity induced by SM.

## 2. Materials and Methods

### 2.1. Chemicals

Sulfometuron-methyl (CAS No. 74222-97-2, purity > 98%) was purchased from Sigma-Aldrich (St. Louis, MO, USA). TransZol Up Plus RNA Kit and cDNA Reverse Transcription Kit were purchased from TransGen Biotech (Beijing, China). SYBR Green system was purchased from Novo Protein (Shanghai, China). Superoxide dismutase (SOD), catalase (CAT), and malondialdehyde (MDA) assay kits were purchased from Nanjing Jiancheng Bioengineering Institute (Nanjing, China). IWR-1 and BML-284 were purchased from MedChem Express (Monmouth Junction, NJ, USA). BCA protein assay kit was purchased from Elabscience Biotechnology (Wuhan, China).

### 2.2. Zebrafish Husbandry and Embryo Collection

Wild-type AB strain zebrafish were obtained from the China Zebrafish Resource Center and maintained in a standalone recirculating aquaculture system (Shanghai Xinjing Biotechnology Co., Ltd., Shanghai, China) under conditions of the physical and chemical parameters of the water used for aquaculture specifically include: pH ranging from 7.2 to 7.6, electrical conductivity of 450–550 μS/cm, dissolved oxygen of ≥6.0 mg/L, as well as the maintained temperature of the aquaculture system (28 ± 0.5 °C) and the photoperiod (14 h of light/10 h of darkness). Adult fish were fed freshly hatched Artemia twice daily. Upon sexual maturity, male and female fish were placed in spawning tanks at a ratio of 1:2 overnight, and fertilized eggs were collected at the onset of light the next morning. Collected embryos were cultured in E3 embryo medium (5 mM NaCl, 0.17 mM KCl, 0.33 mM CaCl_2_, 0.33 mM MgSO_4_, pH 7.4). Developmental stages were determined according to the description of Kimmel et al., expressed as hours post-fertilization (hpf) [22]. All animal experiments were performed in accordance with relevant guidelines and approved by the Institutional Animal Care and Use Committee.

### 2.3. SM Exposure Protocol

A 10 g/L SM stock solution was prepared in DMSO and stored at 4 °C in the dark. Immediately after collection (approximately 1.75 hpf), healthy fertilized embryos were placed in Petri dishes containing E3 medium supplemented with the designated concentrations of SM (0, 10, 20, or 40 mg/L) to initiate exposure [5]. At 6 hpf, the embryos were randomly distributed into 6-well plates (20 embryos per well) containing fresh E3 medium with 0.003% PTU and the corresponding SM concentrations to facilitate observation and subsequent sampling. The exposure was continuously maintained from 1.75 hpf up to 72 hpf. To eliminate the potential confounding effect of solvent concentration, the final DMSO concentration was adjusted to a uniform 0.4% (*v*/*v*) in all treatment groups, including the control group, by adding appropriate amounts of DMSO to the control and lower-concentration groups. Fresh SM solutions were replaced every 24 h, and dead embryos were removed. The stability of SM in the exposure medium was verified by measuring the actual concentrations according to the HPLC-DAD method described previously [5]. The measured concentrations remained within 10% of the nominal values throughout the exposure period. For each independent experiment, three replicate wells (20 embryos per well) were set up for each concentration group. The entire experiment was independently performed three times on different days using different batches of embryos. These three independent experiments served as the biological replicates (*n* = 3) for all subsequent statistical analyses. For each biological replicate, the measurements from the three technical replicate wells were averaged to obtain a single data point.

### 2.4. Developmental Toxicity Assessment

During the exposure period (12, 24, 48, 72 hpf), the numbers of dead and malformed embryos were recorded daily, and cumulative mortality was calculated. Embryonic hatching rate, body length, and yolk sac area were observed and recorded under a stereomicroscope (Leica M205FA, Leica Microsystems, Wetzlar, Germany). Body length was measured as the distance from the head to the tail, and yolk sac area was measured as the lateral projection area of the yolk sac. At least 20 embryos per group were evaluated, and the experiment was independently repeated three times.

### 2.5. Detection of Mitotic Cells

Mitotic cells were labeled by immunostaining for phospho-histone H3 (H3P). To directly evaluate the effect of SM on embryonic cell proliferation, embryos were collected at 12 hpf (gastrula peak, active cell division) and 24 hpf (segmentation period), fixed overnight in 4% paraformaldehyde at 4 °C on a shaker. After gradient methanol dehydration, acetone clearing, and washing with 3% PT (0.1% Triton X-100 in PBS), samples were blocked with PBTN (0.1% Triton X-100 + 5 mg/mL bovine serum albumin + PBS + Tween 20) for 2 h at 4 °C on a shaker, followed by incubation with anti-H3P primary antibody (1:500, Affinity Biosciences, Cincinnati, OH, USA) overnight at 4 °C on a shaker. After washing with 3% PT, samples were counterstained with DAPI (1 μg/mL) for 30 min, washed again, and observed under a laser scanning confocal microscope (Leica TCS SP8, Leica Microsystems, Wetzlar, Germany). The number of H3P-positive cells was counted in whole embryos at 12 and 24 hpf. For each biological replicate, ten embryos per group were randomly selected for H3P-positive cell quantification. All embryos were imaged under a laser scanning confocal microscope (Leica TCS SP8, Wetzlar, Germany) using identical acquisition settings (laser power, gain, offset, and Z-stack step size). To ensure consistent orientation, all embryos were mounted with the dorsal side facing upward, and Z-stack images were acquired from the animal pole to the vegetal pole to capture the entire embryo. The number of H3P-positive nuclei was counted manually using the Cell Counter plugin in ImageJ 1.8.0_172 software (NIH, Bethesda, MD, USA) by a single observer who was blinded to the treatment conditions. To avoid double-counting overlapping cells, each Z-stack image was carefully scrolled through all optical sections, and only nuclei with clearly defined borders and distinct H3P staining were counted; nuclei appearing in multiple adjacent sections were counted only once. The counts from these ten embryos were averaged to obtain a single data point representing that biological replicate. The entire experiment was independently performed three times (*n* = 3 biological replicates).

### 2.6. Oxidative Stress Assay

At 48 hpf, embryos from each group were collected (40 embryos per group), homogenized in 200 μL cold 0.9% saline, centrifuged at 12,000 *g* for 15 min at 4 °C, and the supernatant was collected. Protein concentration was determined using the BCA protein assay kit. SOD activity, CAT activity, and MDA content were measured according to the kit manufacturers’ instructions; MDA level in each sample was calculated based on a standard curve and then normalized to the total protein concentration, which was determined using a BCA protein assay kit. The final MDA content was expressed as nmol per mg of protein (nmol/mg protein). Embryos from each group were collected for each biological replicate. The 40 embryos from the three replicate wells of the same concentration were pooled to prepare one sample. The biochemical assays for SOD, CAT, and MDA for each sample were performed in technical triplicates, and the mean value of these technical replicates was used as a single data point for that biological replicate.

### 2.7. RNA Extraction and Quantitative Real-Time PCR

For each biological replicate, total RNA was extracted from a pool of approximately 50 embryos per group at 72 hpf. RNA concentration and purity (OD260/OD280 ratio between 1.8 and 2.0) were determined using a NanoDrop One spectrophotometer (Thermo Fisher Scientific, Waltham, MA, USA). One microgram of RNA was reverse-transcribed into cDNA using oligo-dT primers and the PrimeScript RT Kit (TransGen Biotech, Beijing, China) according to the manufacturer’s protocol. qRT-PCR was performed using the SYBR Green method on a real-time PCR system (qTOWER3 G, Analytik Jena GmbH, Jena, Germany). β-actin was used as the internal reference gene. Prior to qRT-PCR analysis, the amplification efficiency of each primer pair was determined using a standard curve generated from a 10-fold serial dilution series of pooled cDNA. Each dilution was run in triplicate. All primer pairs used in this study exhibited amplification efficiencies between 90% and 100%, with R^2^ values > 0.98. Subsequently, qRT-PCR was performed in technical triplicates for each sample. The average Ct value from the technical triplicates was used to calculate the relative expression level (2^−ΔΔCt^) for that biological replicate. Primer sequences for target genes are listed in Table S1.

### 2.8. Western Blot

Embryos at 72 hpf (*n* = 60 per group) were collected. Total protein was extracted using RIPA lysis buffer containing protease and phosphatase inhibitors. After SDS-PAGE, proteins were transferred to PVDF membranes. Membranes were blocked with 5% non-fat milk for 2 h at room temperature and then incubated overnight at 4 °C with primary antibodies anti-C-myc (Thermo Fisher Scientific, Waltham, MA, USA; Cat#MA5-12077, 1:100, 49–65 kDa), anti-Axin2 (Affinity Biosciences, Cincinnati, OH, USA; Cat#DF6978, 1:500, 85–95 kDa), anti-Ctnnb2 (Affinity Biosciences, Cincinnati, OH, USA; Cat#AF6267, 1:500, 80–100 kDa), and anti-β-actin (Affinity Biosciences, Cincinnati, OH, USA; Cat#AF7018, 1:500, 43 kDa). After washing with TBST, membranes were incubated with HRP-conjugated secondary antibodies (1:500) for 1 h at room temperature. Signals were visualized by ECL chemiluminescence, and band intensities were quantified using ImageJ 1.8.0_172 software (NIH, Bethesda, MD, USA).

### 2.9. Rescue Experiments

To verify the role of the Wnt/β-catenin signaling pathway in SM developmental toxicity, healthy embryos at 1.75 hpf were randomly divided into four groups: Control (0.4% DMSO), 40 mg/L SM (0.4% DMSO), 40 mg/L SM (0.4% DMSO) + 0.25 μM IWR-1 (Wnt inhibitor), and Control (0.4% DMSO) + 5 nM BML-284 (Wnt activator). After exposure, observe and record the developmental phenotype of the embryos, measure the body length and the area of the yolk sac, and statistically analyze the changes in relevant indicators.

### 2.10. Statistical Analysis

All data are presented as mean ± standard deviation (SD) from at least three independent biological replicates. For each biological replicate, embryos from separate spawning events were used, and all experimental procedures were performed independently. Statistical analyses were performed using GraphPad Prism 8.0 software (GraphPad Software, San Diego, CA, USA). Comparisons among multiple groups were conducted using one-way ANOVA followed by Dunnett’s multiple comparison test. To formally evaluate the concentration-dependent trends, the Jonckheere-Terpstra trend test (for non-parametric data) or a linear trend test (for parametric data) was performed where appropriate. *p*-value < 0.05 was considered statistically significant, with significance levels denoted as * *p* < 0.05, ** *p* < 0.01, and *** *p* < 0.001.

## 3. Results

### 3.1. Exposure to SM Induces Developmental Retardation in Zebrafish Embryos

**Figure 1:**
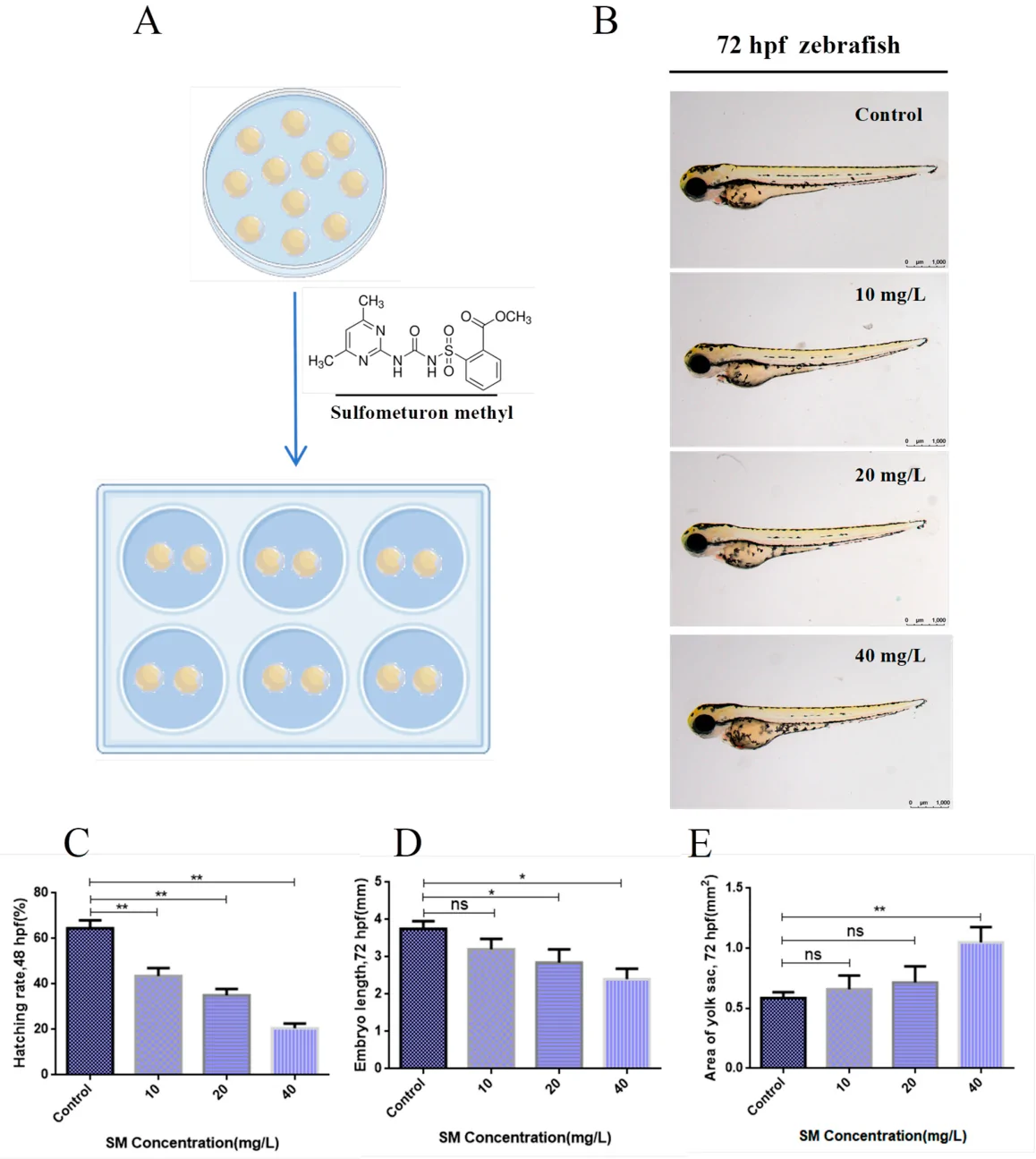
Developmental toxicity of sulfometuron-methyl in zebrafish embryos. (**A**) The molecular formula of SM and the schematic diagram of the zebrafish exposure experiment; (**B**) The phenotype of 72 hpf zebrafish larvae; (**C**) The hatching rate of each group at 48 hpf; (**D**) The body length statistics of larvae at 72 hpf; (**E**) The area of the yolk sac of larvae at 72 hpf (The representative images are selected based on the average body length of each group to best represent the average phenotype. Data are presented as mean ± SD from three independent experiments (*n* = 3 biological replicates). “ns” indicates no significant difference. * *p* < 0.05, ** *p* < 0.01).

To assess the effect of SM on the early embryonic development of zebrafish, we exposed 1.75 hpf embryos to 10, 20, and 40 mg/L SM up to 72 hpf (Figure 1A,B). The results showed that SM exposure significantly reduced the cumulative hatching rate of the embryos in a concentration-dependent manner (Figure 1C; Jonckheere-Terpstra trend test, Z = −4.52, *p* < 0.001). At 48 hpf, the hatching rate of the control group was approximately 62%, while the hatching rates of the 10, 20, and 40 mg/L SM treatment groups decreased to approximately 43%, 31%, and 19%, respectively. Morphologically, the zebrafish larvae in the SM treatment group showed obvious developmental retardation at 72 hpf. Compared with the control group, the body length of the embryos in the SM treatment group was significantly shortened (*p* < 0.05), and the yolk sac area increased (yolk sac edema) (Figure 1D,E). These results indicate that SM exposure can induce developmental retardation in zebrafish embryos, manifested as delayed hatching, retarded growth, and morphological abnormalities.

### 3.2. SM Exposure Reduces Mitotic Active Cells in Early Embryos and Induces Cell Apoptosis

**Figure 2:**
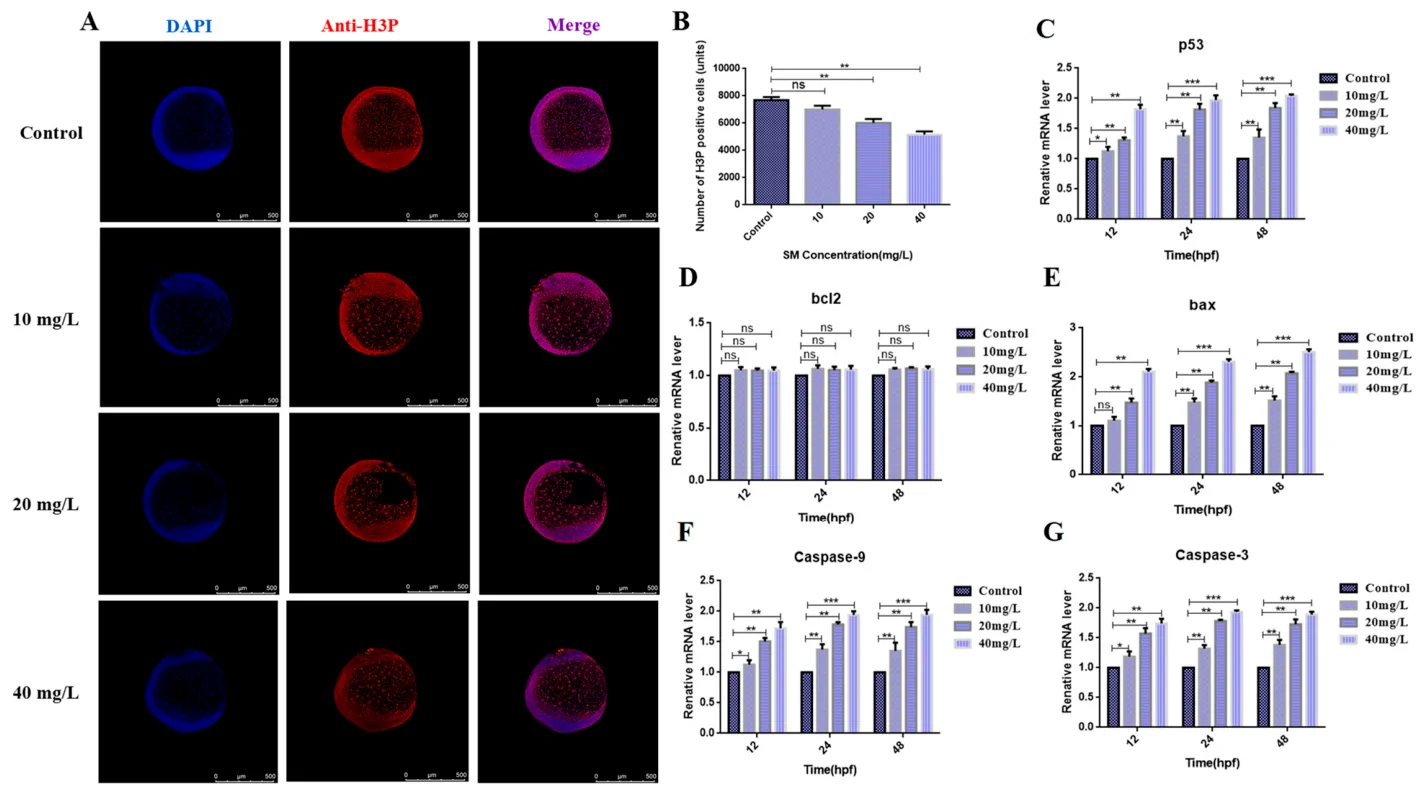
Analysis of mitotic active cells and apoptosis in zebrafish embryos after SM exposure. (**A**) Representative images of H3P immunostaining of embryos at 12 hpf in each group, blue, DAPI; red, H3P; purple, merge; (**B**) Quantitative statistics of H3P-positive cells; (**C**) Relative mRNA expression levels of *p53* in each treatment group; (**D**) Relative mRNA expression levels of *bcl-2* in each treatment group; (**E**) Relative mRNA expression levels of *bax* in each treatment group; (**F**) Relative mRNA expression levels of *caspase-9* in each treatment group; (**G**) Relative mRNA expression levels of *caspase-3* in each treatment group. (Data are presented as mean ± SD from three independent experiments (*n* = 3 biological replicates). For each biological replicate, ten embryos per group were quantified by manual counting of Z-stack images under blinded conditions. “ns” indicates no significant difference. * *p* < 0.05, ** *p* < 0.01, *** *p* < 0.001).

To explore the cytological basis of developmental delay, we used H3P immunofluorescence staining to label mitotic cells. The results showed that compared with the control group, the number of H3P-positive cells in the SM-exposed embryos was significantly reduced in a concentration-dependent manner (Figure 2A; Jonckheere-Terpstra trend test, Z = −4.89, *p* < 0.001). In the 40 mg/L treatment group, the number of H3P-positive cells was approximately 40% less than that of the control group (Figure 2B). This result indicates that SM exposure significantly inhibited the cell mitotic activity in early embryos, which may be the cytological basis for SM-induced embryonic developmental delay and morphological abnormalities.

To determine whether the reduction in cell number involves apoptosis, qRT-PCR was used to detect changes in the expression of related genes. The results showed that compared with the control embryo group, the pro-apoptotic genes *p53* and *bax* were significantly upregulated in the SM-treated group, while the anti-apoptotic gene *bcl-2* showed no significant change (Figure 2C–E); the downstream apoptotic execution genes *caspase-9* and *caspase-3* were also significantly upregulated (Figure 2F,G), especially in the 20 and 40 mg/L concentration groups. These results indicate that SM exposure reduces cell mitotic activity and simultaneously upregulates the transcription of pro-apoptotic genes, suggesting that apoptotic pathways may be initiated. The combined effect of reduced mitotic entry and increased apoptotic signaling likely contributes to the net reduction in embryonic cell numbers.

### 3.3. SM Exposure Induces Oxidative Stress and Apoptosis

**Figure 3:**
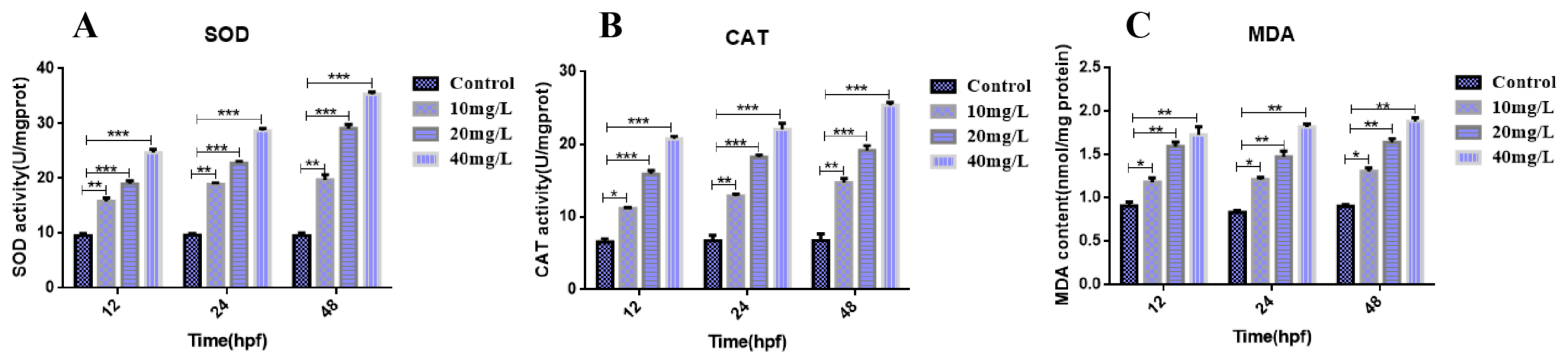
SM exposure induces oxidative stress in zebrafish embryos. (**A**) The activity of SOD in each treatment group; (**B**) the activity of CAT in each treatment group; (**C**) the content of MDA in each treatment group. (Data are presented as mean ± SD from three independent experiments (*n* = 3 biological replicates). * *p* < 0.05, ** *p* < 0.01, *** *p* < 0.001; linear trend test for MDA: F = 9.78, *p* < 0.01).

Oxidative stress is an important upstream trigger for cell apoptosis and proliferation inhibition. We measured the antioxidant enzyme activities in zebrafish embryos exposed to SM. The results showed that the activities of SOD and CAT significantly increased in the SM treatment group (Figure 3A,B), indicating a compensatory dysregulation of the antioxidant defense system in response to SM-induced oxidative challenge. The MDA level showed a concentration-dependent increase (Figure 3C; linear trend test, F = 9.78, *p* < 0.01), suggesting the occurrence of lipid peroxidation. These results, together with the observed upregulation of apoptosis-related genes, suggest that oxidative stress may serve as an upstream trigger for cell apoptosis and contribute to the embryotoxic effects of SM.

### 3.4. SM Exposure Causes Aberrant Expression of Cell Cycle Genes

**Figure 4:**
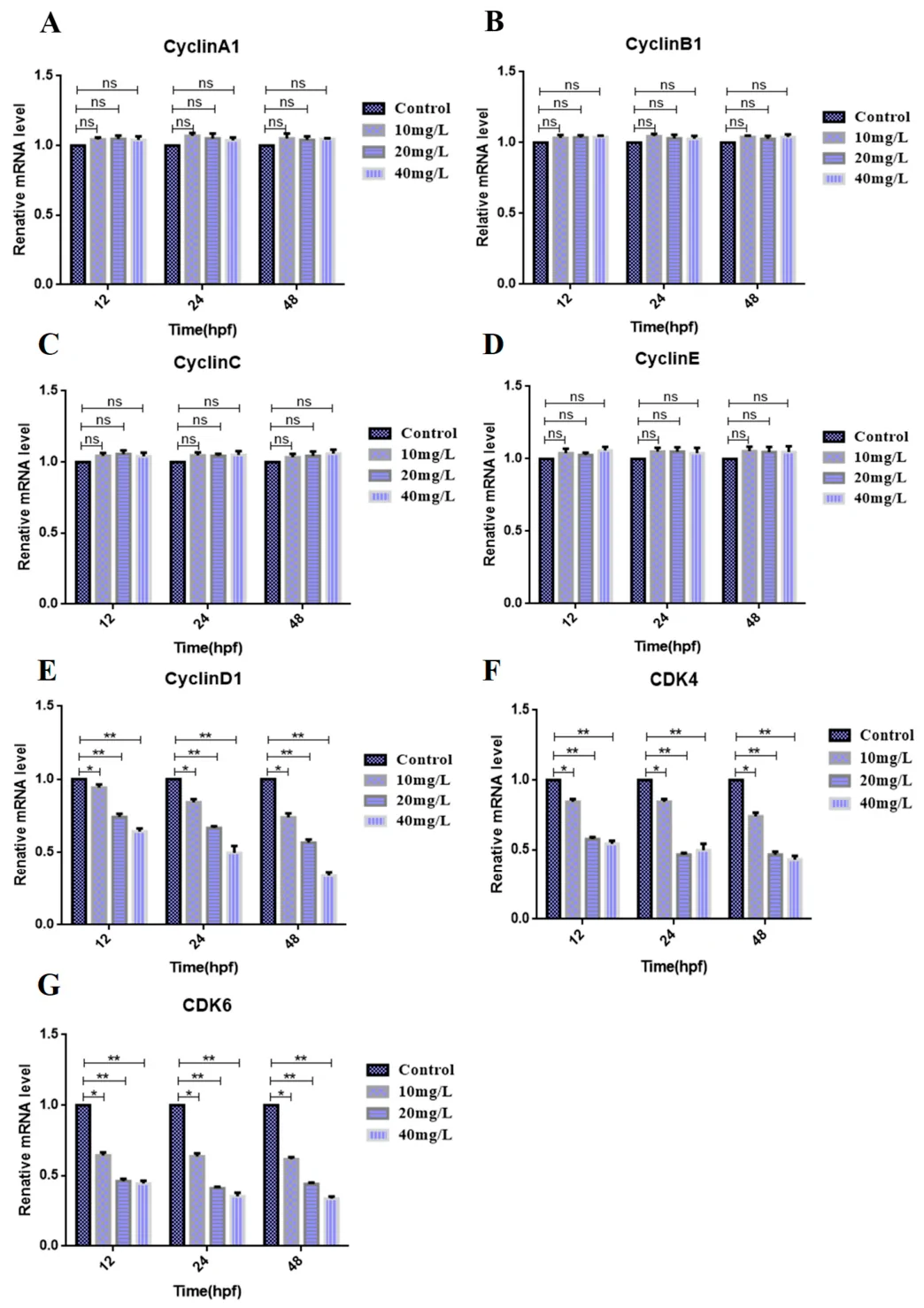
Changes in the expression of cell cycle genes after SM exposure. (**A**) Relative expression level of *CyclinA1*; (**B**) Relative expression level of *CyclinB1*; (**C**) Relative expression level of *CyclinC*; (**D**) Relative expression level of *CyclinE*; (**E**) Relative expression level of *CyclinD1*; (**F**) Relative expression level of *CDK4*; (**G**) Relative expression level of *CDK6*. (Data are presented as mean ± SD from three independent experiments (*n* = 3 biological replicates). “ns” indicates no significant difference. * *p* < 0.05, ** *p* < 0.01).

To further explore the molecular mechanism by which SM affects cell proliferation and apoptosis, this study employed qRT-PCR to detect the transcriptional levels of cell cycle-related genes. The results showed that after exposure to SM, the expression of *CyclinA1*, *CyclinB1*, *CyclinC*, and *CyclinE* did not show significant changes (Figure 4A–D), while the mRNA level of *CyclinD1* exhibited a concentration-dependent significant downregulation. At the same time, the transcriptional levels of the protein kinases *CDK4* and *CDK6*, which functionally bind to *CyclinD1*, also decreased gradually with increasing SM concentration (Figure 4E–G). Given that *CyclinD1* is a well-established direct transcriptional target of the Wnt/β-catenin signaling pathway, its downregulation is consistent with the observed alterations in Wnt pathway components. However, it should be noted that while this correlation is suggestive, the current data do not directly demonstrate that the downregulation of *CyclinD1* is causally driven by Wnt pathway dysregulation. The observed coordinated changes in *CyclinD1*, *CDK4*, and *CDK6* expression indicate that SM exposure negatively affects G1/S-phase regulatory factors, which would be expected to impede cell cycle progression and early embryonic development. Future studies employing pathway-specific modulation would be required to establish a direct causal link.

### 3.5. Exposure to SM Activates the Wnt/β-Catenin Signaling Pathway

**Figure 5:**
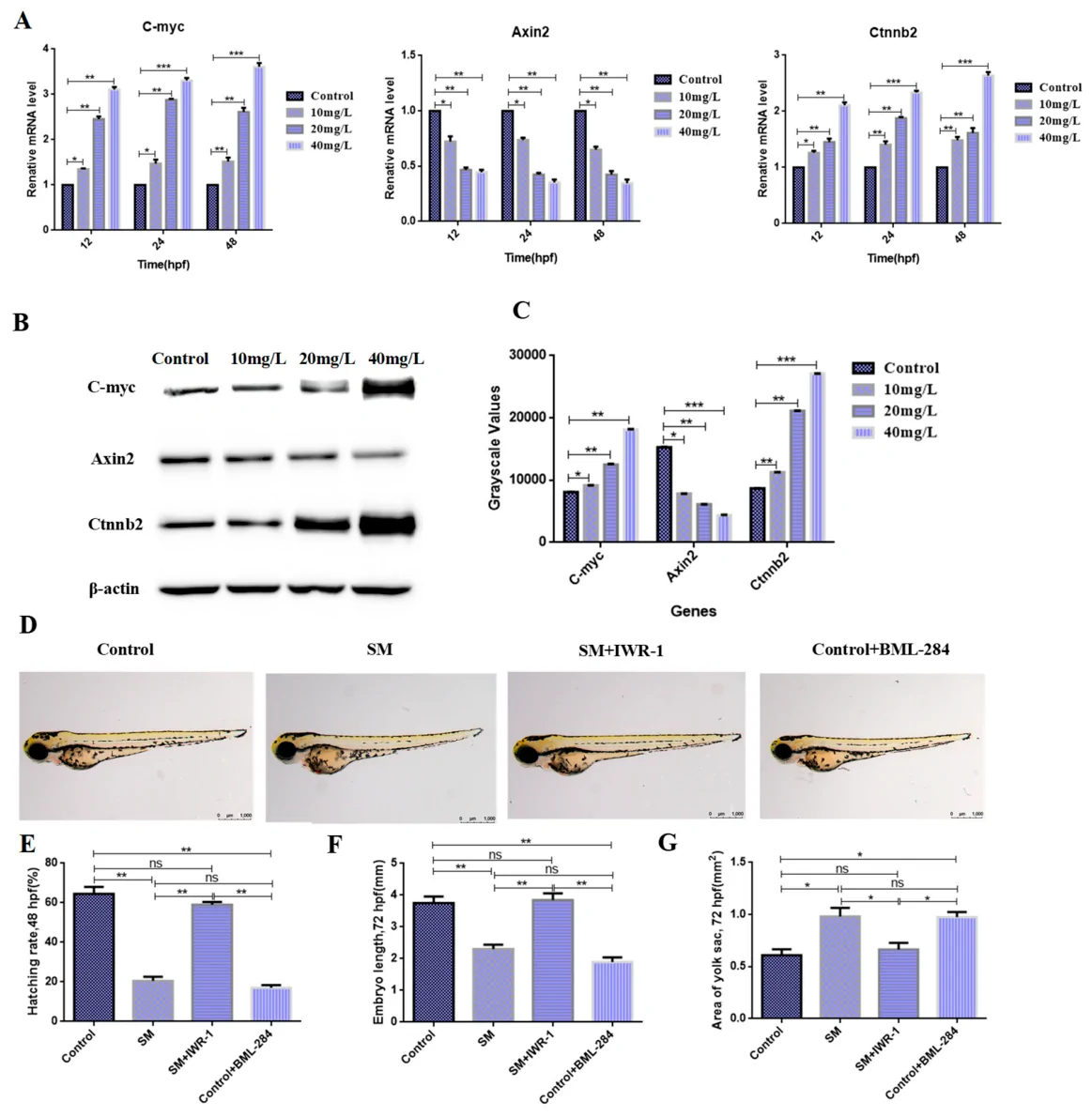
The role of the Wnt/β-catenin signaling pathway dysregulation in the developmental toxicity of SM. (**A**) Changes in mRNA expression of Wnt target genes *C-myc*, *Axin2*, and *Ctnnb2* after SM exposure; (**B**) Changes in protein expression of C-myc, Axin2, and Ctnnb2; (**C**) Gray values of protein expression of C-myc, Axin2, and Ctnnb2; (**D**) Representative morphological images of embryos in the Control group, SM group, SM + IWR-1 group, and Control + BML-284 group; (**E**) Hatch rates of embryos in each group at 48 hpf; (**F**) Body length statistics of embryos in each group at 72 hpf; (**G**) Area of yolk sac in embryos at 72 hpf in each group. (Data are presented as mean ± SD from three independent experiments (*n* = 3 biological replicates). * *p* < 0.05, ** *p* < 0.01, *** *p* < 0.001; ns indicates no significant difference).

To further elucidate the upstream molecular events causing developmental toxicity by SM, based on the core regulatory role of the Wnt/β-catenin signaling pathway in embryonic development and cell proliferation, as well as the molecular characteristics of *CyclinD1* as its direct downstream target gene, this study detected the transcriptional levels of the target genes related to this pathway. The results showed that after SM exposure, the mRNA expression of Wnt target genes *C-myc* and *Ctnnb2* significantly increased in a concentration-dependent manner (Figure 5A; *C-myc*: linear trend test, F = 19.45, *p* < 0.001; *Ctnnb2*: F = 17.32, *p* < 0.001), while the expression of the negative feedback regulator *Axin2* significantly decreased (Figure 5A; linear trend test, F = 21.08, *p* < 0.001), indicating that SM treatment is associated with altered expression of Wnt/β-catenin pathway components, consistent with dysregulation of this signaling cascade in a concentration-dependent manner. The dysregulated state of this pathway is coupled with the inhibition of expression of the aforementioned cell cycle genes, suggesting that the excessive dysregulation of the Wnt signal may be the key upstream molecular event triggering downstream cell cycle disorders and cell proliferation inhibition.

### 3.6. Wnt/β-Catenin Signaling Pathway Inhibitors Rescue Embryonic Development Retardation Caused by SM

To verify the causal effect of excessive dysregulation of the Wnt/β-catenin pathway in SM-induced developmental abnormalities, this study conducted a functional rescue experiment using the pathway-specific inhibitor IWR-1. Embryos were simultaneously exposed to 40 mg/L SM and 0.25 μM IWR-1 for 72 hpf. The results showed that IWR-1 could significantly reverse the developmental retardation phenotype induced by SM: compared with the SM alone treatment group, the embryonic body length in the combined treatment group was significantly increased, and the abnormally enlarged yolk sac area was significantly reduced (Figure 5D–F), and the overall developmental status tended to be normal compared to the control group. To further confirm the pathogenic role of this pathway, this study also treated wild-type embryos with the Wnt pathway agonist BML-284, and the results showed that it presented a developmental defect phenotype similar to that of the SM treatment group, with no statistically significant differences in body length, yolk sac area, or hatching rate between the BML-284 and SM treatment groups (Figure 5D–G; ns indicates no significant difference). The results of the positive and negative genetic pharmacological intervention confirm each other, strongly suggesting that aberrant dysregulation of the Wnt/β-catenin signaling pathway is a key event in SM-induced developmental toxicity. The ability of IWR-1 to effectively rescue the SM-induced phenotypes further supports the functional involvement of this pathway in the toxic process.

## 4. Discussion

As a representative variety of sulfonylurea herbicides, SM has been widely used in agricultural production due to its efficient herbicidal activity, but the potential risks posed by its environmental residues to aquatic ecosystems have attracted increasing attention [2]. Although there have been studies on the residual behavior and degradation dynamics of SM in soil and crops, there is still a lack of systematic understanding of its toxic effects on aquatic organisms, especially fish in the early development stage, and the internal molecular mechanism [3]. In recent years, SM has been used in the invasion management of Spartina alterniflora in some coastal areas of our country, the resulting ecological security problem of non-target Marine organisms further highlights the urgency of carrying out relevant risk assessment [4]. It should be acknowledged that the SM concentrations employed in the present study (10–40 mg/L) are considerably higher than the environmentally relevant levels reported in surface waters (up to 0.020 μg/L) [5]. This concentration discrepancy is common in mechanism-oriented toxicological studies, where higher doses are often necessary to elucidate the full spectrum of molecular pathways and to identify potential adverse outcome pathways. Nevertheless, the present findings provide important mechanistic insights into SM-induced developmental toxicity and highlight the need for further chronic toxicity studies at environmentally realistic concentrations to better inform ecological risk assessments. In this study, we systematically evaluated the toxicity spectrum of SM on early embryonic development using zebrafish as a model organism and revealed for the first time the critical mediating role of Wnt/β-catenin signaling in its developmental toxicity.

Phenotypic analysis showed that SM exposure significantly reduced the hatching rate of zebrafish embryos in a concentration-dependent manner, and induced typical developmental malformations such as shortened body length and yolk sac edema. The above phenotypic characteristics are highly similar to the developmental toxicity spectrum induced by a variety of sulfonylurea herbicides in zebrafish embryos. For example, pyrazosulfuron-ethyl resulted in delayed yolk absorption, delayed head development, and ocular deformities [13]. Imazosulfuron exposure can cause acute embryonic lethality within 48 h, accompanied by pericardial edema and spinal curvature [14]. Sulfentrazone can also interfere with yolk sac absorption and disrupt the hatching process [23]. This evidence suggests that the developmental toxicity of sulfonylurea herbicides to fish embryos may have a common molecular basis, and the reduction in mitotic activity may be the key cytological event. To test this hypothesis, we examined mitotic activity by H3P immunofluorescence staining. H3P (phosphorylated histone H3) is a well-established marker of cells undergoing mitosis, specifically labeling chromosomes during the M phase of the cell cycle. SM exposure significantly reduced the number of H3P-positive cells in embryos in a concentration-dependent manner, directly reflecting diminished mitotic activity, i.e., fewer cells entering or progressing through M phase. However, it should be noted that a reduction in mitotic cells does not necessarily equate to a global inhibition of cell proliferation, as other factors—such as accelerated progression through M phase, altered cell cycle distribution, or increased cell death—could also contribute to decreased H3P-positive cell numbers. Furthermore, it is important to consider whether the reduced number of H3P-positive cells could be partially attributed to the overall reduction in embryo size or developmental delay caused by SM, rather than solely reflecting a specific effect on cell proliferation. As demonstrated in our phenotypic analyses (Figure 1), SM exposure indeed caused significant developmental retardation, including shortened body length and delayed hatching, which inevitably results in smaller embryos with fewer total cells at the same chronological time point. Notably, the reduction in H3P-positive cells (approximately 40% at the highest concentration) was more pronounced than the reduction in body length (approximately 15–20%), suggesting that the decrease in mitotic cells exceeds what would be expected from size reduction alone. Nevertheless, when interpreted together with our qRT-PCR data showing significant downregulation of G1/S-phase regulators (*CyclinD1*, *CDK4*, and *CDK6*), the reduction in mitotic cells strongly suggests a cell cycle arrest at the G1/S transition, which would consequently impede cell proliferation and contribute to the observed developmental retardation. We acknowledge that the contribution of general developmental delay to the reduced mitotic index cannot be entirely excluded, and future studies employing transgenic proliferation reporters or EdU incorporation assays could provide more direct evidence by normalizing H3P-positive cell numbers to total cell numbers or embryo volume.

Oxidative stress is an important early event in embryotoxicity induced by environmental pollutants. The present study found the increased activities of SOD and CAT observed in SM-treated embryos are indicative of a compensatory upregulation of the primary antioxidant defense system. SOD catalyzes the dismutation of superoxide radicals to hydrogen peroxide, while CAT further detoxifies H_2_O_2_ to water and oxygen. The elevation of these enzymatic activities likely reflects a cellular adaptive response to increased ROS production, rather than constituting direct evidence of oxidative damage per se. Indeed, an increase in antioxidant enzyme activity alone does not necessarily indicate that oxidative damage has occurred; it may instead represent a successful defense response that prevents or limits macromolecular injury. Nevertheless, the concurrent elevation of MDA levels, a byproduct of lipid peroxidation, provides more direct evidence that ROS generation exceeded the scavenging capacity of the antioxidant system, resulting in oxidative damage to cellular membranes. Collectively, these findings suggest that SM exposure induces an oxidative challenge that activates compensatory antioxidant defenses, and when this defense is insufficient, leads to lipid peroxidation and subsequent apoptosis. This pattern fits well with the toxicity mechanisms of a variety of pesticides. For example, Buprofezin can induce developmental toxicity of zebrafish embryos through oxidative stress inducing mitochondria-mediated apoptosis pathway [24]. Tefluthrin exposure can significantly induce apoptosis of larval cells, accompanied by transcriptional dysregulation of apoptosis-related genes (*bcl-2*, *bax*, *p53*, *caspase-3*) [25]. Oryzalin induces mitochondrial dysfunction and apoptosis cascade through ROS production [26]. The above studies collectively suggest that the oxidative stress-apoptosis cascade is a ubiquitous pathogenic axis in the developmental toxicity of pesticides.

At the level of apoptosis, SM exposure significantly up-regulated the transcriptional levels of *p53*, *bax*, *caspase-9* and *caspase-3*, while *bcl-2* expression did not change significantly, resulting in an increased *bax*/*bcl-2* ratio. This molecular signature suggests that SM may trigger apoptosis via a *p53*-dependent mitochondrial endogenous apoptotic pathway. As the guardian of genomic stability, *p53* can be activated in response to DNA damage or oxidative stress, and then transcriptionally up-regulate *bax* and other pro-apoptotic genes, and initiate the caspase cascade to finally execute cell apoptosis. It is worth pointing out that the expression of the anti-apoptotic gene *bcl-2* was not significantly changed in this study, while the pro-apoptotic signal was significantly enhanced, suggesting that SM-induced apoptosis may be mainly achieved by enhancing the signal output of the pro-apoptotic pathway rather than by inhibiting the function of the anti-apoptotic system. However, it should be acknowledged that these conclusions are primarily based on transcriptional changes in key regulatory genes as assessed by qRT-PCR. While these genes are well-established components of the intrinsic apoptotic pathway, changes in mRNA expression levels alone do not definitively demonstrate that the apoptotic program has been executed at the protein or cellular level. Transcriptional upregulation indicates that cells have initiated a transcriptional response consistent with apoptosis, but post-transcriptional regulation, protein stability, and enzymatic dysregulation (particularly of caspases) are also critical determinants of actual cell death outcomes. As emphasized by Martino and Chiarelli [27], direct detection of DNA fragmentation via TUNEL assay or caspase activity/cleaved *caspase-3* detection provides more definitive evidence of apoptosis execution. Nevertheless, the concerted upregulation of multiple pro-apoptotic genes—including the upstream regulator *p53*, the pro-apoptotic *bcl-2* family member *bax*, and the effector caspases *caspase-9* and *caspase-3*—provides coherent transcriptional evidence that the intrinsic apoptotic pathway is initiated in SM-treated embryos. The concurrent downregulation of G1/S-phase cell cycle genes further supports the notion that SM exposure disrupts the balance between cell proliferation and programmed cell death at the transcriptional level. Future studies employing protein-level analyses would be valuable to confirm the dysregulation of apoptosis at the functional level. Similarly, tetrachlorantraniliprole induces neurodevelopmental toxicity in zebrafish through oxidative stress-mediated apoptosis and Wnt signaling pathway dysregulation [19]. Chlorpyrifos can interfere with the mRNA expression of *C-myc*, *Cyclin D1*, *bax* and *bcl-2*, which are closely related to proliferation and apoptosis in embryos [28]. The above findings further support the general notion that pesticides impede normal embryonic development by interfering with apoptotic pathways.

Wnt/β-catenin signaling pathway plays an irreplaceable role in the regulation of vertebrate embryonic development, participating in key processes such as dorsolateral axis specialization, gastrulation, and dorsal neuralization [15]. In this study, SM exposure resulted in significant upregulation of the Wnt target genes *C-myc* and *Ctnnb2*, alongside significant downregulation of *CyclinD1* and the negative feedback regulator *Axin2* (Figure 4E). The interpretation of Wnt/β-catenin pathway dysregulation is supported by both transcriptional and protein-level evidence: qRT-PCR revealed the aforementioned changes in mRNA expression, while Western blot analysis confirmed increased expression of C-myc and Ctnnb2, and decreased expression of Axin2, in SM-treated embryos. These coordinated changes across both mRNA and protein levels provide convergent evidence consistent with pathway dysregulation. It should be noted, however, that while the concurrent upregulation of positive regulators (*C-myc*, *Ctnnb2*) and downregulation of the negative regulator *Axin2* are well-established signatures of activated Wnt/β-catenin signaling, these changes alone do not constitute definitive proof of pathway dysregulation in the absence of functional assays. Nevertheless, the consistency between our transcriptional and protein data, together with the rescue effect of the Wnt inhibitor IWR-1, strongly supports the conclusion that SM exposure leads to aberrant dysregulation of the Wnt/β-catenin pathway.

These results echo those reported in studies of various environmental pollutants. For example, benzophenone exposure can also induce cardiac developmental toxicity by upregulating Wnt signaling [20]. It is worth noting that different pollutants may regulate the Wnt pathway in different directions. Diclofop-methyl exposure can downregulate Wnt signaling [21], whereas oxadiazon-butachlor inhibits this pathway and downregulates its target genes (*lef1*, *axin2*, *β-catenin*) [29]. These differences may be due to different chemicals acting on different nodes of the signaling network or interfering with different upstream regulatory links. It may also reflect the complexity of different chemicals inducing different adaptive responses in the same pathway. The dysregulation effect of SM on Wnt signaling in this study suggests that SM may interfere with normal embryonic development by breaking the fine balance of Wnt signaling in space and time.

To further assess the functional significance of Wnt/β-catenin pathway in SM-induced developmental toxicity, we conducted a bidirectional pharmacological intervention with the pathway-specific inhibitor IWR-1 and agonist BML-284. The results showed that IWR-1 could effectively rescue the embryonic developmental retardation caused by SM, while BML-284 alone could mimic the developmental defect phenotype of SM. This experimental strategy of positive dysregulation and reverse inhibition corroborating each other strongly supports the hypothesis that SM interferes with normal embryonic development through abnormal dysregulation of Wnt/β-catenin signaling. Over-dysregulation of Wnt signaling may disturb the finely orchestrated balance of cell proliferation and differentiation during embryonic development, and eventually lead to abnormal morphogenesis. However, several caveats should be considered when interpreting these pharmacological data. First, small-molecule inhibitors and agonists such as IWR-1 and BML-284 may exert off-target effects that could independently influence embryonic development. It should be explicitly acknowledged that the rescue experiment did not include an IWR-1-only control group. Therefore, we cannot exclude the possibility that IWR-1 alone may have independent effects on embryonic development under our experimental conditions. Importantly, the effects of IWR-1 may depend on the specific experimental conditions (e.g., concentration, exposure window, zebrafish strain, and environmental factors), and conditions reported in the literature may not be directly transferable to our study. Thus, the absence of an IWR-1-only control is a limitation of the present study, and the possibility that IWR-1 independently influences the measured endpoints cannot be entirely excluded. Future studies incorporating an IWR-1-only control group, as well as additional concentrations and exposure windows, will be necessary to more rigorously establish the specificity of the pharmacological rescue. Second, pharmacological intervention demonstrates that modulating this pathway is sufficient to alter the SM-induced phenotype, but it does not definitively establish that Wnt/β-catenin signaling is the sole or primary initiating mechanism of SM toxicity. Other signaling pathways—such as those involved in oxidative stress response, cell cycle regulation, or other developmental signaling cascades—may also contribute to the observed effects and could interact with Wnt signaling in complex ways. Similarly, the proposed cascade in which Wnt dysregulation leads to G1/S cell cycle arrest via *CyclinD1* downregulation, as well as the connection between Wnt dysregulation and oxidative stress/apoptosis, is inferred from correlative evidence obtained at different experimental time points rather than from direct causal experiments. These proposed connections should therefore be viewed as a mechanistic hypothesis that is strongly supported by the current data, but requires further experimental validation.

Nevertheless, the convergence of evidence from transcriptional changes, protein-level alterations, and bidirectional pharmacological rescue—together with the established roles of Wnt signaling in cell cycle regulation and apoptosis—provides a coherent framework for understanding the molecular basis of SM-induced developmental toxicity. Wnt signaling dysregulation has also been identified as one of the major drivers of neurodevelopmental defects in studies of other environmental contaminants, such as tetrachlorantraniliprole [19]. Collectively, these results point to Wnt/β-catenin signaling as a highly conserved and functionally important target in the developmental toxicity of environmental pollutants.

At the level of cell cycle regulation, SM exposure significantly down-regulated the transcriptional levels of *CyclinD1*, *CDK4* and *CDK6.* Among them, *CyclinD1* is the key rate-limiting factor of G1/S phase transition, and its down-regulation can directly lead to cell cycle arrest and proliferation inhibition, which is highly consistent with the decrease in mitotic activity reflected by H3P staining. It is worth noting that *CyclinD1* is a direct transcriptional target gene of the Wnt/β-catenin signaling pathway, and its down-regulation is likely to be a secondary event downstream of abnormal dysregulation of Wnt signaling [30]. Therefore, a clear molecular pathway chain can be concluded: SM exposure → abnormal dysregulation of Wnt/β-catenin signaling → inhibition of ccnd1 and other cell cycle regulatory genes expression → arrest of G1/S phase transition → inhibition of cell proliferation → retardation of embryonic development. However, it is necessary to emphasize that the mechanism proposed in this study—linking the dysregulation of the Wnt pathway with the downregulation of *CyclinD1* expression, G1/S phase arrest, and reduced proliferation—has not yet been experimentally confirmed to have a direct causal relationship between these steps. In the future, we will design relevant experiments to verify this. This mechanism logic is similar to the results of pyridaben, which also showed abnormal expression of *CyclinD1* after exposure and then interfered with the cell cycle order of early embryos [31]. In addition, it is worth emphasizing that the cell cycle gene changes observed in this study were concentrated at the G1/S phase regulatory node (*CyclinD1*/*CDK4*/*CDK6*). However, there were no significant changes in G2/M phase-related genes (*CyclinA1*, *CyclinB1*) and early G1 phase-related genes (*CyclinC*, *CyclinE*), which further suggested that the interference of SM on the cell cycle was node-specific rather than global cycle blocking.

It is important to contextualize our findings relative to the previous study by Yuan et al., which comprehensively characterized the toxic effects of SM on early zebrafish development [5]. That study demonstrated that SM exposure (10–40 mg/L, from 5.5 to 72 hpf) induces developmental toxicity (increased mortality, reduced hatching), immunotoxicity, neurobehavioral alterations, oxidative stress, and apoptosis. While these phenotypic effects are largely consistent with our observations, the present study provides several novel mechanistic insights that were not previously explored. First, we extend the exposure window to cover earlier developmental stages (from 1.75 hpf), allowing assessment of effects on early mitotic activity during gastrulation. Second, we demonstrate for the first time that SM exposure significantly reduces mitotic activity in early embryos, as quantified by H3P-positive cell counts, providing a cytological basis for the observed developmental retardation. Third, we provide the first evidence that SM exposure activates the Wnt/β-catenin signaling pathway, as demonstrated by coordinated upregulation of *C-myc* and *Ctnnb2* and downregulation of *Axin2* at both transcriptional and protein levels. Fourth, we establish a functional link between Wnt pathway dysregulation and SM-induced developmental toxicity through bidirectional pharmacological rescue experiments using the specific inhibitor IWR-1 and agonist BML-284. Fifth, we reveal that SM exposure leads to G1/S-phase cell cycle arrest through downregulation of *CyclinD1*, *CDK4*, and *CDK6*, connecting Wnt pathway dysregulation to impaired cell proliferation. Collectively, these findings provide a mechanistic framework—involving Wnt/β-catenin signaling dysregulation, oxidative stress, cell cycle arrest, and reduced mitotic activity—that substantially advances our understanding of SM developmental toxicity beyond the phenotypic characterization previously reported.

**Figure 6:**
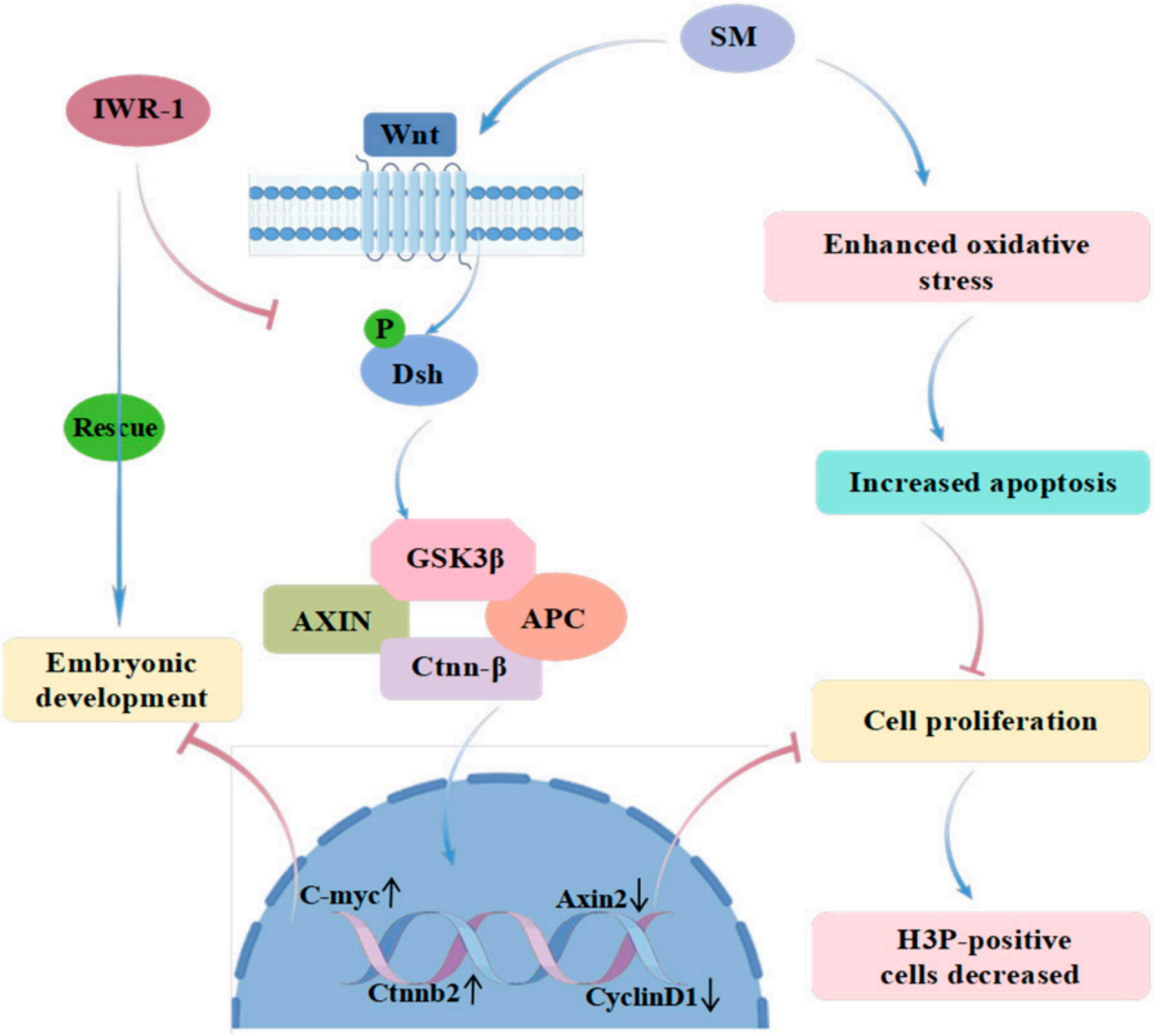
Schematic diagram of the molecular mechanism by which SM affects early zebrafish embryonic development. Exposure to SM dysregulates the Wnt/β-catenin signaling pathway (*C-myc*↑, *Ctnnb2*↑, *Axin2*↓, *CyclinD1*↓), simultaneously inducing oxidative stress and cell apoptosis, reducing mitotic activity (H3P positive cells↓), and ultimately suppressing embryonic development. The Wnt inhibitor IWR-1 can rescue the developmental toxicity of SM. This model represents a synthesis of the observed changes and is proposed as a working hypothesis based on the combined evidence presented in this study.

In summary, the present study provides evidence that SM exposure dysregulates Wnt/β-catenin signaling and induces oxidative stress, transcriptional changes consistent with apoptosis initiation, and reduced mitotic activity in zebrafish embryos (Figure 6). However, it should be emphasized that the proposed cascade linking Wnt pathway dysregulation to oxidative stress, apoptosis, and reduced mitotic activity is primarily based on correlative evidence obtained from independent experiments at different time points, rather than direct causal demonstration. In particular, we did not demonstrate that Wnt activation directly induces oxidative stress or mitochondrial apoptosis, as these endpoints were measured independently and were not assessed following Wnt pathway modulation. Future studies incorporating combined assessments of oxidative stress and cell cycle markers in the context of Wnt pathway modulation would be valuable to establish a direct causal link. This finding not only provides a new molecular perspective for understanding the developmental toxicity of SM, but also enriches the key experimental basis for the aquatic ecological risk assessment of sulfonylurea herbicides. It should be pointed out that this study mainly verified the key nodes of the signaling pathway at the transcriptional level and protein level. Future studies can further combine with gene knockout or conditional overexpression models to more accurately verify the causal role of the Wnt/β-catenin pathway at the genetic level. In addition, the long-term exposure effects of SM at different environmental concentrations and the risk of transmission through the food chain need to be further explored. In view of the extensive residue and potential bioaccumulation of SM in the environment, as well as its new application scenario for S. alterniflora management in coastal areas of China, the aquatic ecological risks of SM need to be included in a broader perspective of monitoring and management, and the relevant environmental standards and use regulations should also be reevaluated in time.

## 5. Conclusions

In this study, we systematically evaluated the toxic effects of SM on early embryonic development in zebrafish and elucidated the molecular mechanisms involved. The main conclusions are as follows: SM exposure induced typical developmental toxic phenotypes of zebrafish embryos in a concentration-dependent manner, including reduced hatching rate, shortened body length, and yolk sac edema, confirming the developmental damage effects of SM. SM exposure significantly reduced the number of mitotically active cells in embryos, indicating that cell proliferation arrest was an important cytological basis for developmental retardation. SM exposure induced an oxidative challenge in embryos, as evidenced by compensatory increases in SOD and CAT activities and elevated MDA levels, suggesting that oxidative stress and subsequent lipid peroxidation are involved in the toxic process of SM. SM exposure resulted in the down-regulation of cell cycle-regulating key genes (*CyclinD1*/*CDK4*/*CDK6*) and the up-regulation of apoptosis-related genes (*p53*, *bax*, *caspase-3*, *caspase-9*), which revealed the transcriptomic signatures of SM interfering with cell cycle progression and initiating apoptotic signaling at the molecular level. SM exposure abnormally dysregulates the Wnt/β-catenin signaling pathway, and the specific inhibitor IWR-1 of this pathway can effectively rescue the developmental retardation phenotype caused by SM, which strongly supports that excessive dysregulation of the Wnt/β-catenin signaling pathway plays an important functional role in the developmental toxicity of SM.

In summary, this study established a molecular link between SM exposure and abnormal dysregulation of Wnt/β-catenin signaling, and provides a coherent framework for understanding the molecular basis of “signaling pathway-oxidative stress-cell cycle arrest/apoptosis-reduced mitotic activity-developmental toxicity”. This finding not only provided a new scientific basis and molecular target for the aquatic ecological risk assessment of SM, but also opened up a new perspective for the ecotoxicology study of sulfonylurea herbicide residues, suggesting that the potential aquatic ecological risks of SM should be given full attention in environmental monitoring and management policies. It should be noted that the absence of an IWR-1-only control group is a limitation of this study, and future experiments including this control will be important to confirm the specificity of the rescue effects.

## Data Availability

The original contributions presented in this study are included in the article/Supplementary Material. Further inquiries can be directed to the corresponding author.

## Supplementary Materials

The following supporting information can be downloaded at https://www.mdpi.com/article/10.3390/toxics14090835/s1. Table S1 lists qRT-PCR primer sequences.
