# Supplementary material for: Sulfometuron-Methyl Disrupts Early Zebrafish Embryonic Development via Wnt/β-Catenin Signaling Pathway
Source: Toxics. 2026 Sep 20;14(9):835. doi: 10.3390/toxics14090835 (PMC13611569; doi:10.3390/toxics14090835)
Supplement: Supplementary file 1 [file toxics-14-00835-s001.zip › toxics-4523621-supplementary.pdf]

Table S1 lists qRT-PCR primers sequences

Gene	Sequence	Efficiency (%)
bax q-PCR FP	TCGAACATGATCTGTGTCATC	99.1
bax q-PCR RP	TATGGCTGGGGTCACTTTTCTC	
bcl-2 q-PCR FP	TGGCGTCCCAGGTAGATAAT	96.8
bcl-2 q-PCR RP	ACCGTACATCTCCACGAAGG	
p53 q-PCR FP	CCCGGATGGAGATAACTTG	97.2
p53 q-PCR RP	CACAGTTGTCCATTCAGCAC	
Caspase-3 qPCR-fp	GAGACCGCTGCCCATCACTAG	98.0
Caspase-3 qPCR-rp	ATCCTTTCACGACCATCT	
Caspase-9 qPCR-fp	GCCTTTCTTGATTCCCTGCG	97.5
Caspase-9 PCR-rp	TCTTGGCCTGGTTGGTCTCA	
Cyclin A1 qPCR-fp	CGGCCAAAGAATTGTGCTCG	95.3
Cyclin A1 qPCR-rp	TGAAGGCAGCAGGAATGATGT	
Cyclin B1 qPCR-fp	GCAGTTGTAGCGAACAAGCC	96.3
Cyclin B1 qPCR-rp	GGAAGGCTCAGACACAACCT	
Cyclin C qPCR-fp	AGTGGGTTCTGGACAAGCAG	97.6
Cyclin C qPCR-rp	GGGCCATTAACACAGGGTCA	
Cyclin D1 qPCR-fp	TGCTGCGAAGTGGATACCAT	95.8
Cyclin D1 qPCR-rp	GCTCCTAAAAGCTGCAACCTG	
Cyclin E qPCR-fp	CGCAGTATGCATCAGAAAGC	97.1
Cyclin E qPCR-rp	TCCATAACGCGTGTATCTCG	
CDK4 qPCR-fp	CAATCAGGACGGACTCCCAC	96.1
CDK4 qPCR-rp	ATTAGTGTGCAGGAACGCCA	
CDK6 qPCR-fp	CAGCTCGAGTCCTTTGAGCA	97.8
CDK6 qPCR-rp	CGATGAACCACACGGTGAGA	
C-myc qPCR-fp	GAAAGCCGGAGTTCAGCCTA	98.2
C-myc qPCR-rp	TCAGACTGACCCCCAGAAAGA	
Axin2 qPCR-fp	CTCCCCACCTTGAATGAAGA	96.6
Axin2 qPCR-rp	ACTGGGTCGCTTCTCTTGAA	
Ctnnb2 qPCR-fp	GGCTACAGACAGGAAGACCCA	97.4
Ctnnb2 qPCR-rp	AACCAGGCCAGTTGGTTGGAG	
β-actin q-PCR FP	AGCACGGTATTGTGACTAACTG	98.5
β-actin q-PCR RP	TCGAACATGATCTGTGTCATC	
